# Human Metapneumovirus: Epidemiology and genotype diversity in children and adult patients with respiratory infection in Córdoba, Argentina

**DOI:** 10.1371/journal.pone.0244093

**Published:** 2020-12-28

**Authors:** Pamela Elizabeth Rodriguez, María Celia Frutos, María Pilar Adamo, Cecilia Cuffini, Jorge Augusto Cámara, María Gabriela Paglini, Laura Moreno, Alicia Cámara

**Affiliations:** 1 Instituto de Virología “Dr. J. M. Vanella”, Facultad de Ciencias Médicas, Universidad Nacional de Córdoba, Córdoba Capital, Córdoba, Argentina; 2 Instituto de Investigaciones Médicas Mercedes y Martín Ferreyra, INIMEC- CONICET, Universidad Nacional de Córdoba, Córdoba Capital, Córdoba, Argentina; 3 Cátedra de Clínica Pediátrica, Facultad de Ciencias Médicas, Universidad Nacional de Córdoba, Hospital de Niños “Santísima Trinidad de Córdoba”, Córdoba Capital, Córdoba, Argentina; Defense Threat Reduction Agency, UNITED STATES

## Abstract

Human Metapneumovirus (hMPV) is responsible for acute respiratory infections in humans, with clinical and epidemiological relevance in pediatric, elderly, and immunocompromised populations. These features are largely unknown in Córdoba, Argentina and in adults in general. Hence, our goal was to broadly characterize hMPV infection in patients of all ages hospitalized with acute respiratory infections in Córdoba, Argentina, including epidemiology, clinical features and genetic diversity. Nasopharyngeal secretions were obtained from 795 patients during 2011–2013, 621 patients were 0–25 years old and 174 were 26–85 years old. HMPV was assayed by RT-PCR and other respiratory viruses by indirect immunofluorescence. Local strains were identified by sequence analysis. Human Metapneumovirus was detected in 20.3% (161/795) patients, 13.1% as single infections and 7.2% in co-infections, more frequently with Respiratory Syncytial Virus. HMPV circulated during late winter and spring in all age patients, but mainly in children under 4 years old in 71.4% (115/161) and adults between 26 and 59 years old in 12.4% (20/161). The most prevalent diagnosis was mild acute respiratory infection in 59.6% (96/161) and bronchiolitis in 9.3% (15/161). Local strains were clustered within A_2_ subtype; they presented 73–100% identities among them, showing a high degree of homology compared to isolations from neighboring countries. We demonstrate that hMPV circulated among all age patients with respiratory infection during 2011–2013 in Córdoba, contributing to the understanding of this virus, its diagnosis and patient handling in local health-care centers.

## Introduction

Viruses responsible for acute respiratory infections (ARI) have a great impact on human health and continue to emerge; in consequence, their diagnosis and surveillance constitute a milestone for the Public Health system. Human Metapneumovirus (hMPV- *Pneumoviridae* family) was isolated for the first time by Van den Hoogen in The Netherlands (2001), who detected it in respiratory samples of children younger than 5 years of age [1, 2].

The hMPV spherical particle has an envelope and its genome is constituted by a negative single-stranded and not segmented RNA of 13.3-Kb long. It has 8 genes that encode for 9 proteins: nucleoprotein (N), phosphoprotein (P) large protein (L), matrix proteins (M, M2_1_, M2_2_), and glycoproteins G (adhesion), F (fusion) and SH (small hydrophobic) [3–5].

Severe ARI caused by hMPV leading to hospitalization usually occur in children under 5 years are detected in 5–15% of the cases, representing the second most frequent viral cause of ARI after respiratory syncytial virus (RSV). Primary infection with hMPV occurs during the first 2 years of life and re-infections can occur repeatedly until adulthood [6, 7]. The infection can be subclinical or associated with bronchiolitis, pneumonia and asthma exacerbation, especially in children. In children, hMPV infection can cause fever, runny nose, cough, pharyngitis, otitis, wheezing and hypoxia [5, 8].

The virus also infects 4–5% of young adults, producing generally mild infections with symptoms such as cough, nasal congestion, hoarseness, sore throat and fever [7, 8]. Nevertheless, older adults and immunocompromised patients may present more severe clinical manifestations [5] and those with chronic diseases constitute also a risk group since they can undergo severe hMPV infection (4–13%), sometimes associated with fatal outcomes [5, 9, 10].

HMPV has been detected in single infections and in co-infections with other respiratory pathogens, including rhinoviruses, RSV, parainfluenza virus (PIV), influenza (Flu), bocavirus, and coronavirus [5, 7, 11].

This agent has a seasonal distribution; peaks have been reported at the end of winter and during spring, after RSV and Flu. In temperate regions, hMPV co-circulates with PIV during winter and until late spring [5, 8].

Phylogenetic analyses have demonstrated two hMPV genotypes: A and B; they are classified into sub-genotypes 1 and 2 (A_1_, A_2_, B_1_, B_2_) [12]. The amino acid sequence of F protein shows 94–97% identity when comparing A and B lineages, and less than 98% between the subtypes (A_1_ vs. A_2_ and B_1_ vs. B_2_), while the N gene is more conserved when comparing all subgenotypes (91.2% and 98.4%) [7, 8, 13, 14]. Both genotypes can circulate simultaneously; the predominance of one or the other depends on the year and region. It has also been proposed that different genotypes may produce infections with different degrees of severity [7, 8].

HMPV is widely distributed worldwide [8]. In Buenos Aires, Argentina, it has been detected in 11% of children with ARI and subtypes A_1_, A_2_, and B_1_ were identified [15, 16]. In contrast, in an early study carried out in our laboratory, in Córdoba, hMPV was detected in 4% of pediatric patients hospitalized with lower ARI [17]. In addition, the general epidemiology of the virus and its impact in adults and the elderly is still unknown in Córdoba, where it is not generally included in the differential diagnosis of respiratory diseases.

Here we describe epidemiological features, clinical, and molecular diversity of hMPV infecting patients of all ages hospitalized due to ARI during 2011–3013 in Córdoba, Argentina. We demonstrate that human Metapneumovirus circulated in all age patients during this period.

## Materials and methods

### Ethical considerations

This study complied with the principles outlined by the Declaration of Helsinki and was approved by an independent Ethics Committee of Children Hospital "Santísima Trinidad" (CIEIS Protocol: 05/2011). All participants included or their parents/legal guardians signed a written informed consent prior to clinical specimen collection and the data were analyzed anonymously.

### Study design and specimen collection

A prospective descriptive study was performed. Inclusion criteria: patients of all ages hospitalized with signs or symptoms related to ARI and/or a diagnosis of ARI, subjects from Córdoba (capital of Córdoba province), and hospitalized from January 2011 through December 2013. Exclusion criteria were patients with ambulatory treatment and hospitalization for other causes than ARI.

The patients were evaluated by the physiologists, who considered their inclusion in the study and invited them to participate. Clinical respiratory specimens were obtained by nasopharyngeal aspirate (NPA) or nasopharyngeal swab (NPS) according to age. The samples were obtained from eight different hospitals of Córdoba.

Demographic, epidemiological, and clinical data of patients were recorded in ad-hoc forms, including date, age, gender, preexisting medical conditions, signs and symptoms at the time of hospitalization, and clinical diagnosis. The samples were collected according to established clinical protocols and then delivered on ice to the Laboratory of Respiratory Viruses, Instituto de Virología "Dr. J. M. Vanella", Facultad de Ciencias Médicas, Universidad Nacional de Córdoba, for analysis.

### Diagnostic panel for respiratory viruses

Samples were tested for Flu A and B, RSV, Adenovirus (AdV), and PIV 1–3 using an indirect immunofluorescence (IF) assay (LIGHT DIAGNOSTICS^™^ Respiratory Viral Panel I Screening Kit & Identification IFA. Temecula, CA, USA) according to the manufacturer’s instructions. This is used for routine laboratory diagnosis in the clinical practice.

### Human Metapneumovirus detection

Nucleic acids from all samples were extracted with the QIAamp^®^ Viral RNA Mini Kit (Qiagen, Hilden, Germany) following the manufacturer instructions. PCR for detection of hMPV N gene was used to amplify a 199 bp fragment, using the Qiagen OneStep RT-PCR kit (Qiagen, Hilden, Germany). The protocol for conventional RT-PCR was adapted from Bouscambert-Duchamp [18]. This PCR was validated for hMPV diagnosis and was implemented in our laboratory. We used the following hMPV primers: Fw 5´-GTGATGCACTCAAGAGATACCC-3´ and Rv 5´- CATTGTTTGACCGGCCCCATAA-3´, 50 μM each. Cycling conditions: 30 min at 50°C and 15 min at 94°C, followed by 40 cycles of 95°C for 30 sec, 58°C for 30 sec and 72°C for 1 min, plus one final step of 72°C for 10 min. PCR products were separated by electrophoresis on a 1.5% agarose gel and visualized under UV light after ethidium bromide staining.

### Human Metapneumovirus genotyping

RT-PCR protocol was adapted from Van den Hoogen [19] to amplify a 696 bp fragment of F gene, using the Qiagen OneStep RT-PCR kit (Qiagen, Hilden, Germany), with primer mix Fw 5´-CAATGCAGGTATAACACCAGCAATATC-3´ and Rv 5´-GCAACAATTGAACTGATCTTCAGGAAAC-3´, 50 𝜇M each. Cycling conditions: 30 min at 42°C and 8 min at 95°C, followed by 40 cycles of 94°C for 1 min, 40°C for 2 min and 72°C for 3 min, and a final step of 72°C for 10 min. The amplified products were separated by electrophoresis on a 1.5% agarose gel and visualized under UV light after ethidium bromide staining.

All PCR products were purified with QIAquick Gel Extraction Kit (Qiagen, Hilden, Germany) according to the manufacturer’s instructions. Human MPV RNA high quality for sequencing was selected. The nucleoprotein gene region (199 bp) and the fusion gene (696 bp) were subjected to direct nucleotide sequencing reactions in both directions using the internal PCR primers by Macrogen, Inc. (Seoul, Korea).

The sequences obtained were edited with MEGA 4.0.2 [20] and independent alignments were made for N and F gene with sequences available in the GenBank, using ClustalW.

Maximum likelihood trees (ML) were constructed with the PhyML 3.1 software [21]. The branch support was evaluated via non-parametric bootstrapping with 1000 pseudoreplicates. The nucleotide substitution model for each data set analyzed was selected according to the Akaike information criteria implemented in the ModelTest 3.7 software [22].

The sequences used for phylogenetic analysis (GenBank) for N and F genes are shown in S1 Table. Sequences shorter than 200 bp (N gene) are available in S2 Table. F gene sequences were deposited in GenBank (accession nos. MN136292, MN136293, MN136294, and MN136295).

### Data analysis and graphics

Data and graphics were prepared with Graph Pad Prism 5.0 (GraphPad Software, San Diego, California, USA). The prevalence was expressed as percentage. The seasonal distribution of hMPV, RSV, Flu A- B, AdV and PIV 1–3, was organized according to months (2011–2013). For the age distribution of cases, patients were classified into 6 age-groups, which resulted in the following sample sizes: ≤12 months = 378; 13 months to 4 years = 124; 5–14 years = 89; 15–25 years = 30; 26–59 years = 128 and ≥ 60 years = 46. Categorical variables were compared using the Two-tailed Chi-square test. P-value of <0.05 was considered statistically significant.

## Results

In total, 795 patients ranging from 0 to 85 years old were enrolled in this study. Of them, 53.84% (428/795) were male, and 46.16% (367/795) female. The median age was 12 months (IQR: 2–132), 621 samples corresponded to patients 0–25 years old and 174 samples to patients 26–85 years old.

### Human Metapneumovirus prevalence

Of the 795 clinical specimens analyzed, 52.8% (420/795) resulted positive for respiratory viruses (hMPV, Flu-A, Flu-B, RSV, AdV, and PIV 1–3), either in single or co-infections. Human Metapneumovirus occurred in a ratio of 72:89 female to male patients, respectively. The prevalence of hMPV was 20.3% (161/795), of which 65% (104/161) were single infections and 35% (57/161) were double infections. Among double infections, the most frequent co-infecting agent was RSV (35/57). RSV was the most prevalent virus, followed by hMPV and Flu-A (Table 1).

**Table 1:** Viral detection in hospitalized patients in single and co-infections (N = 795).

HOSPITALIZED		
	n	% Prevalence (n = 795)
**Virus detected**	359	45.2
**Single infection**		
RSV	141	17.7
hMPV	104	13.1
Flu-A	99	12.5
PIV 3	8	1
Flu-B	3	0.4
AdV	2	0.3
PIV 1	2	0.3
**Double infection**	61	7.7
hMPV- RSV	35	4.4
hMPV- Flu-A	16	2.0
hMPV- PIV 3	3	0.4
hMPV- AdV	1	0.1
hMPV- Flu-B	2	0.3
RSV-PIV 3	2	0.3
RSV- Flu A	2	0.3

hMPV: Human Metapneumovirus; Flu A: Influenza Virus A; Flu B: Influenza Virus B; RSV: Respiratory Syncytial Virus; AdV: Adenovirus; PIV: Parainfluenza Virus.

### Age distribution of hMPV cases

Human Metapneumovirus was detected in all age-groups studied. The median age of hMPV positive patients was 12 month (IQR = 2–111), while hMPV was most prevalent in the group of infants under 12 months (24.34%, 92/378), followed by patients 15–25 years (20%, 6/30), children 13 months to 4 years old (18.55%, 23/124) and 5–14 years old (16.85%, 15/89).

In addition, it is worth noting that 15.62% (20/128) cases occurred in patients 26–59 years old and 10.86% (5/46) were adults older than 60 years.

The most prevalent virus was RSV and 98% of the RSV positive samples corresponded to children under 4 years of age. However, Flu A was more frequent in patients older than 15 years.

### Seasonal distribution of hMPV

**Fig 1:**
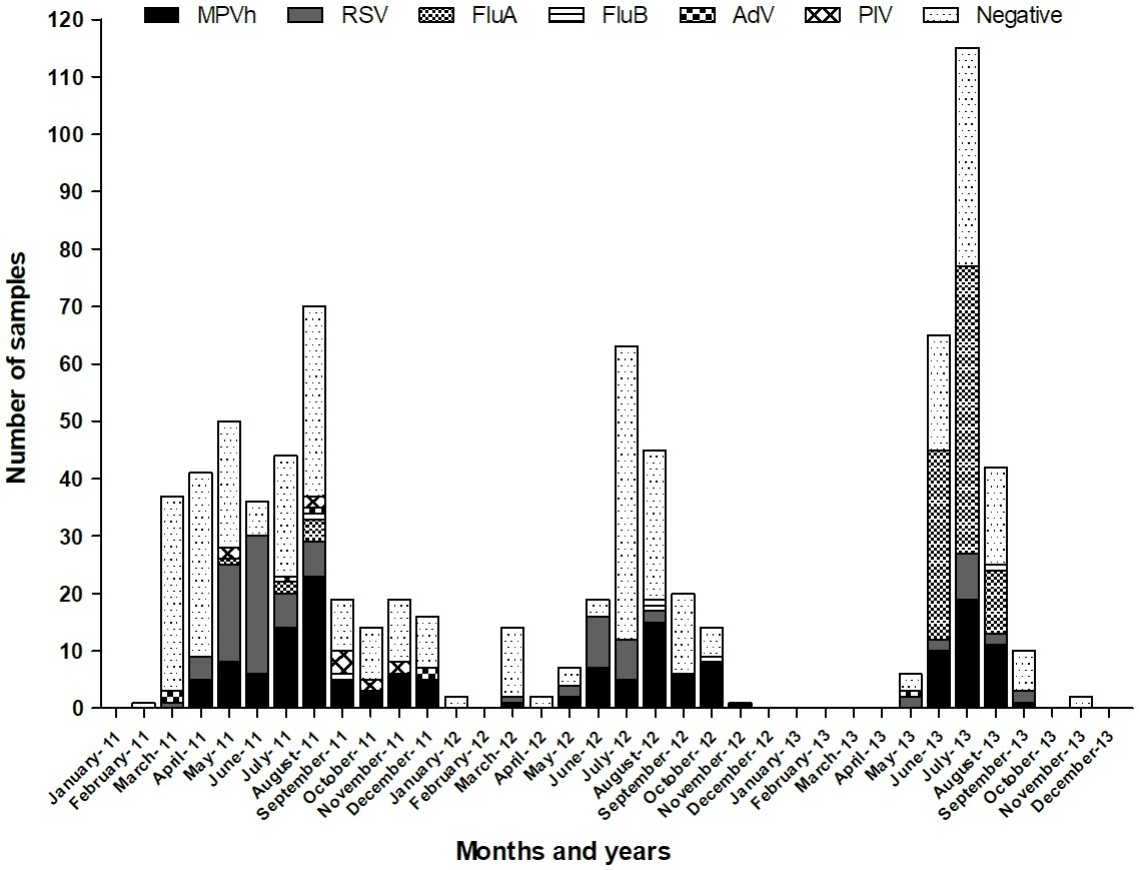
Seasonal distribution of hMPV. Seasonal distribution of hMPV, RSV, Flu A, Flu B, AdV, PIV 1–3 during 2011–2013 in Córdoba, Argentina. The distribution is sorted by month. (n = 795). hMPV: Human Metapneumovirus. Flu: Influenza. AdV: Adenovirus. PIV: Parainfluenza virus.

Throughout the three years studied, hMPV circulated from autumn to late spring with a prevalence peak between July and August. As shown in Fig 1, hMPV circulation extended during the spring months, after the winter outbreaks of Flu A and RSV. In 2011 and 2012 hMPV cases were detected even during November and December (late spring).

### Clinical presentation of hMPV in single and co-infection

The most frequent signs/symptoms in all patients infected with respiratory viruses were fever, cough, sore throat, headache, and weakening. In hMPV positive patients the most frequent symptoms were fever, cough, and headache.

Regarding the clinical evaluation of hMPV infected patients, 59.6% (96/161) were hospitalized for breathing difficulty and with a diagnosis of mild ARI. Other frequent respiratory diseases were bronchiolitis in 9.31% (15/161) cases and pneumonia of viral etiology in 3.72% (6/161). Also, 4.35% (7/161) hMPV infected patients were admitted for respiratory difficulty syndrome (RDS) and recurrent bronchial obstruction (RBO), and 3.72% (6/161) for asthmatic crisis or asthma exacerbation. We compared the clinical presentation of patients with hMPV single detection versus those with hMPV detected in co-infection with other respiratory viruses; the only difference we found was the diagnosis of bronchitis present in co-infected patients (p = 0.0182) (Table 2).

**Table 2:** Admitting diagnosis of 161 patients with hMPV single and co-infection.

Diagnosis at admission	Total hMPV (n = 161)	Single hMPV (n = 104)	hMPV in co-infection (n = 57)	*p-value*
**Mild ARI**	96 (59.6)	62 (59.6)	34 (59.6)	0.9967
**Bronchiolitis**	15 (9.3)	7 (6.7)	8 (14)	0.1273
**Bronchitis**	3 (1.83)	0 (0)	3 (5.3)	0.0182
**Pneumonia**	6 (3.7)	3 (2.9)	3 (5.3)	0.4461
**RDS**	7 (4.3)	5 (4.8)	2 (3.5)	0.6991
**RBO**	7 (4.3)	6 (5.8)	1 (1.75)	0.2322
**Asthmatic crisis/Asthma exacerbation**	6 (3.7)	4 (3.8)	2 (3.5)	0.9139
**Influenza-like illness**	6 (3.7)	2 (1.9)	4 (7)	0.1027
**NA**	15 (9.3)	15 (14.4)	0 (0)	-

hMPV: human Metapneumovirus; ARI: Acute respiratory infection; RDS: Respiratory difficulty syndrome; RBO: Recurrent bronchial obstruction; NA: Not available data.

Mild ARI was the most frequent presentation in all age groups analyzed. Bronchiolitis, RDS, and RBO were diagnosed in children less than 12 months and asthma exacerbation occurred in children between 1–14 years. HMPV in single infection was significantly more prevalent in infants ≤12 month (p = 0.0007) and hMPV in co-infection was significantly more prevalent in the 26–59 age group (p = 0.0439). In pediatric patients comorbidities were not presented. No severe complications or deaths were related to hMPV.

### Human Metapneumovirus genotypes

Optimal nucleotide sequences were obtained from 11 hMPV positive samples, 7 from N gene and 4 from F gene. Table 3 shows the clinical and epidemiological features of patients whose samples were sequenced.

**Table 3:** Demographic and clinical features of 11 patients with hMPV infection whose samples were sequenced and genotyped.

Strain ID	Gender	Age^a^	Gene	Sample date	Clinical presentation
Cordoba/ARG/2590/2011	M	6	N	April/2011	Mild ARI
Cordoba/ARG/2629/2011	F	8	N	May/ 2011	Mild ARI
Cordoba/ARG/2708/2011	F	24	N	June/2011	RDS
Cordoba/ARG/2768/2011	M	79	F	July/2011	Mild ARI
Cordoba/ARG/2732/2011	M	24	N	June/2011	Mild ARI
Cordoba/ARG/2755/2011	M	1	N	July/2011	Mild ARI
Cordoba/ARG/2840/2011	F	6	N	August/2011	Mild ARI
Cordoba/ARG/2895/2011	F	6	F	August/2011	RBO
Cordoba/ARG/2881/2011	F	5	F	August/2011	Mild ARI
Cordoba/ARG/2899/2011	M	5	N	August/2011	Mild ARI
Cordoba/ARG/3064/2011	M	3	F	December/2011	Pneumonia

All samples were nasopharyngeal swabs. ARI: Acute respiratory infection; RDS: Respiratory difficulty syndrome; RBO: Recurrent bronchial obstruction.

^a^Month.

**Fig 2:**
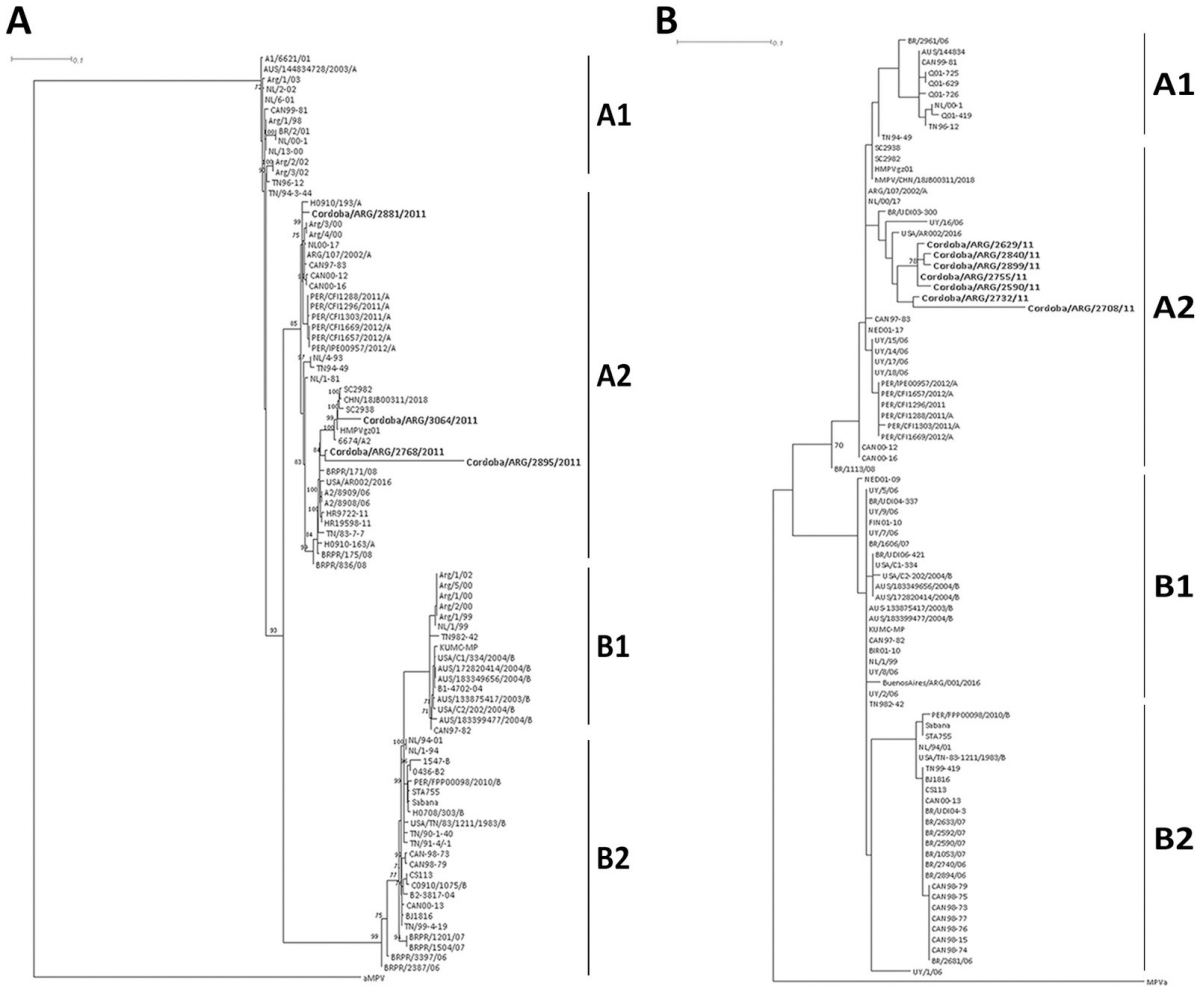
Phylogenetic analysis of human metapneumovirus based on F and N genes. (A) F gene. A Maximum Likelihood tree was constructed using the GTR+G model with parameters suggested by JModelTest 3.7 with bootstraps and 1000 pseudoreplicates. (B) N gene. A Maximum Likelihood tree was constructed using the TIM3+G model with parameters suggested by JModelTest 3.7 with bootstraps and 1000 pseudoreplicates. A1: strains to subgroup A_1_ of hMPV. A2: strains to subgroup A_2_ of hMPV. B1: strains to subgroup B_1_ of hMPV. B2: strains to subgroup B_2_ of hMPV. Avian MPV (aMPV) was used to root the tree. The strains of interest are marked in bold. The scale bar indicates the changes between nucleotides.

Fig 2A shows ML tree based on F gene fragment, where the local strains analyzed were clustered in hMPV A_2_ subtype, together with sequences previously reported in Argentina. The Cordoba/ARG/2881/2011 strain is highly related to prototype strains from Canada, The Netherlands, and Argentina. Cordoba/ARG/3064/2011 strains were more related to isolates from the United States, China, and Nepal. Cordoba/ARG/2768/2011 and Cordoba/ARG/2895/2011 strains are closely related to each other, but Cordoba/ARG/2895/2011 presented more nucleotide changes with an identity of 83% compared to the other local strains, which had 96–100% identity among them.

Fig 2B shows ML tree based on N gene fragment. The Córdoba strains also were identified as hMPV A_2_ and grouped with sequences from America and other regions. Cordoba/ARG/2590/2011, Cordoba/ARG/2629/2011, Cordoba/ARG/2708/2011, Cordoba/ARG/2732/2011, Cordoba/ARG/2755/2011, Cordoba/ARG/2840/2011, and Cordoba/ARG/2899/2011 strains were clustered in one group related to strains from Brazil, Uruguay, and the United States. Cordoba/ARG/2708/2011 strain presented 72–76% homology compared to other strains from Cordoba identified in this study, which had 92–97% identity among them.

## Discussion

This study describes for the first time the molecular epidemiology and genetic diversity of hMPV infection and circulation in Cordoba, Argentina, during the years 2011–2013. This agent caused ARI in children and adult patients and we detected 13.1% (104/795) hMPV as single infection. Also, hMPV was detected in co-infections with other viruses in 7.2% (57/795) of the patients. In terms of prevalence, hMPV was the second most frequently detected virus after RSV (22.6%). Mild ARI were more frequent in all our groups studied. As reported by other authors, in developing countries, ARI has a great impact on morbidity in the general population and on morbidity/mortality in children and immunocompromised patients [23]. For this reason, viral infections such as hMPV responsible as causative agents of an important proportion of ARI, need to be studied, surveyed, treated and controlled, as they are associated with a substantial economic burden caused by hospitalizations and outpatient visits [24, 25].

Generally, hMPV prevalence varies between 4 and 22%, depending on the age group and detection method [6, 19, 26, 27] however, few studies include elderly patients. In our study, hMPV was detected in all age groups, being more prevalent in infants less than 12 month old (24.3%) and among patients 15–26 years old (20%). Velez Rueda (2013) found similar prevalence in the pediatric population in our country [28], while it was detected in 11% of the children under 5 years of age in the first report of hMPV in Argentina [15]. Other countries including Mexico, Spain, and Brazil, have reported higher prevalence in the pediatric population, reaching 20–25% [29–32]. Van den Hoogen (2003), reported higher prevalence in children younger than 5 years old and detected cases in all age groups, even in subjects over 65 years old. In other countries, hMPV was detected in prevalence between 4 and 5% in adults 25–50 years old [30, 33]. Thus, hMPV is a frequent agent of ARI in subjects of all ages and re-infections are common in children younger than 5 years of age, different than RSV, which is more common in infants and children under 2 years [30, 34].

During the three years studied, we observed that hMPV had a higher incidence in August 2011 and 2012 and July of 2013, lasting until late spring. Thus, in Córdoba, hMPV is a prevalent respiratory agent in frank winter season, with cases occurring from the beginning of the cold season throughout the spring. Human Metapneumovirus infections occur throughout the year but the highest frequency of cases in the southern hemisphere occurs after the epidemic peak of RSV [8]. Several studies highlight the typical distribution of this virus in early spring, while circulation during summer seems to depend on local weather conditions [11, 15, 35, 36].

In this study, co-infection of hMPV with other viruses was detected in 35% (57/161) of cases, along with RSV (61%), Flu-A (28%), PIV (5%), Flu-B (4%), and AdV (2%). These results are in agreement with previous publications, in which a 35%-80% hMPV- RSV co-infection rate has been reported, due to similar seasonal distribution [19, 35].

With respect to the clinical presentation, the most common symptoms in our study were fever, cough, and headache and the most frequent diseases were mild ARI, bronchiolitis, pneumonia, RDS, and RBO. Other authors have reported similar findings [30, 34, 35, 37] although those studies also report symptoms like wheezing and dyspnea. Mild ARI was frequent in all age groups studied, more prevalent in infants under 12 months. Generally, in the pediatric population, the most frequent clinical presentations are bronchiolitis and pneumonia [15, 26, 28, 36]; in our study bronchiolitis only occurred in infants under 12 months. We found cases of hMPV infection associated to asthma exacerbation in children under 14 years. This is important because other authors have proposed that asthma exacerbation should be more related to hMPV in comparison to RSV, mainly due to recurrent infections with different virus subtypes [38], and hMPV infection was indeed associated with severe RBO as was described in other studies [39].

Since the discovery of hMPV, two genotypes have been demonstrated: A and B [1]. We identified the hMPV A_2_ subtype and our results showed that it circulated in Córdoba during 2011. We did not detect subtypes A1, B1 and B2; a reason for this may have been we were not able to get high-quality RNA and/or optimal sequences from the years 2012 and 2013, however, other authors demonstrated that these subtypes are seldom reported [40]. In our country, Galiano (2006) demonstrated the circulation of A_1_, A_2_ and B_2_ subtypes; Velez Rueda (2013) showed that A_2_ and B_2_ subtypes co-circulated during 2009–2010, while hMPV A_2_ was detected in 2011 [16, 28]. The local strains showed high homology to prototype strains from The Netherlands, Canada, Argentina [16], and neighboring countries such as Uruguay [41], Brazil [42, 43], and Peru [44]. The high homology among some strains (93–100% identity) compared to other strains (73–83% identity) from Córdoba identified in this study, indicates co-circulation of strains with different genetic variability, which would predispose hMPV reinfections.

Limitations of our study need to be considered. This study was not including detection of bacterial agents, human bocavirus, human coronaviruses, enteroviruses, and rhinoviruses. In addition, the routine laboratory diagnosis in the clinical practice is still performed by immunoflorescence assay, which is a technique with lower sensitivity compared to PCR (used here for the detection of hMPV). On the other hand, balancing with this issue, we should mention that the extremely high sensitivity of molecular methods inflict complexity to assigning the etiological agent in many cases, as multiple co-infections are frequent due to the ability of these techniques to detect the smallest amounts of nucleic acid of infecting agents. Taking all of these into account, our analysis of the signs and symptoms associated to hMPV infection is useful as an approximation to further focus on characterizing the clinical presentation of this virus in single infection and when the viral load is high.

This study is the first report of hMPV in Córdoba. As we have shown a high prevalence in hospitalized patients, we contribute to improving the diagnosis and treatment of acute respiratory infections by recommending its local routine detection and inclusion of hMPV in the respiratory diagnostic panel. In this way we also contribute to the surveillance of respiratory agents. These data allow us to expand our knowledge on hMPV in Córdoba, Argentina, and future studies are justified to elucidate the clinical presentation of the infection and thus contribute to a fitter handling of patients.

## Supporting information

S1 Table Sequences available in the GenBank for both the N and F genes used for phylogenetic analysis. (DOC)

S2 Table Local sequences of N gene shorter than 200 bp. (DOC)
