# Supplementary material for: Human Metapneumovirus: Epidemiology and genotype diversity in children and adult patients with respiratory infection in Córdoba, Argentina
Source: PLoS One. 2020 Dec 28;15(12):e0244093. doi: 10.1371/journal.pone.0244093 (PMC7769284; doi:10.1371/journal.pone.0244093)
Supplement: S1 Table — (DOC) [file pone.0244093.s001.doc]

**S1Table. Sequences available in the GenBank for both the N and F genes used for phylogenetic analysis.**

| **HMPV sequence name** | **Country** | **GenBank access number** | **Reference** |
| --- | --- | --- | --- |
| HMPV/ARG/107/2002/A | Argentina | KF686742.1 | [1] |
| HMPV/BuenosAires/ARG/001/2016 | Argentina | MG773272.1 | [2] |
| STA755 | Brazil | MG431250.1 | [3] |
| NL/00/1 | Netherlands | NC_039199.1 | [4] |
| NL/17/00 | Netherlands | AY296012 | [5] |
| NL/1/99 | Netherlands | AY304360 | [5] |
| NL/1/94 | Netherlands | AY304362 | [5] |
| NL/17/00 | Netherlands | AY304360.1 | [5] |
| NL/00/17 | Netherlands | FJ168779.1 | [6] |
| NL/94/01 | Netherlands | FJ168778.1 | [6] |
| TN96-12 | United States | JN184399.1 | [7] |
| TN982-42 | United States | JN184401.1 | [7] |
| TN94-49 | United States | JN184400.1 | [7] |
| TN/99/4-19 | United States | JN184402.1 | [7] |
| TN/99/4-19 | United States | EU857581.1 | [8] |
| Sabana | Rwanda | HM197719.1 | [9] |
| HMPV/USA/C1-334/2004/B | United States | KC562242.1 | [1] |
| HMPV/USA/C2-202/2004/B | United States | KC562235.1 | [1] |
| HMPV/USA/TN-83-1211/1983/B | United States | KC562244.1 | [1] |
| hMPV/USA/AR002/2016 | United States | KY474530.1 | [10] |
| HMPV/AUS/144834728/2003/A | Australia | KC562241.1 | [1] |
| HMPV/AUS/133875417/2003/B | Australia | KF530179.1 | [1] |
| HMPV/AUS/183399477/2004/B | Australia | KF530163.1 | [1] |
| HMPV/AUS/183349656/2004/B | Australia | KF530173.1 | [1] |
| HMPV/AUS/172820414/2004/B | Australia | KF530167.1 | [1] |
| CAN98-73 | Canada | AY145279.1-AY145287.1 | [11] |
| CAN97-82 | Canada | AY145277.1-AY145295.1 | [11] |
| CAN98-75 | Canada | AY297748.1 | [12] |
| CAN98-75 | Canada | AY145289.1 | [11] |
| CAN98-79 | Canada | AY145285.1-AY145293.1 | [11] |
| CAN97-83 | Canada | AY145278.1-AY145296.1 | [11] |
| CAN00-12 | Canada | AY145272.1-AY145297.1 | [11] |
| CAN00-13 | Canada | AY145273.1-AY145298.1 | [11] |
| CAN00-16 | Canada | AY145276.1-AY145301.1 | [11] |
| PER/CFI1296/2011/A | Peru | KJ627388.1 | [13] |
| PER/CFI1288/2011/A | Peru | KJ627407.1 | [13] |
| PER/FPP00544/2011/A | Peru | KJ627416.1 | [13] |
| PER/CFI1303/2011/A | Peru | KJ627425.1 | [13] |
| PER/CFI1657/2012/A | Peru | KJ627423.1 | [13] |
| PER/IPE00957/2012/A | Peru | KJ627433.1 | [13] |
| PER/CFI1669/2012/A | Peru | KJ627413.1 | [13] |
| hMPV-CHN-18JB00311-2018 | China | MK087726.1 | [14] |
| KUMC-MP | South Korea | KF516922.1 | [15] |
| CS113 | China | EF081361.1-EF081369.1 | [16] |
| HMPVgz01 | China | GQ153651.1 | [17] |
| BJ1816 | China | DQ843658.1 | [18] |
| aMPV | Brazil | MF093139.1 | [19] |

| **HMPV (F) sequence name** | **Country** | **GenBank access number** | **Reference** |
| --- | --- | --- | --- |
| Arg/2/02 | Argentina | DQ362937.1 | [20] |
| Arg/3/02 | Argentina | DQ362938.1 | [20] |
| Arg/1/98 | Argentina | DQ362939.1 | [20] |
| Arg/1/03 | Argentina | DQ362940.1 | [20] |
| Arg/3/00 | Argentina | DQ362941.1 | [20] |
| Arg/4/00 | Argentina | DQ362942.1 | [20] |
| Arg/1/02 | Argentina | DQ362943.1 | [20] |
| Arg/1/99 | Argentina | DQ362944.1 | [20] |
| Arg/5/00 | Argentina | DQ362945.1 | [20] |
| Arg/2/00 | Argentina | DQ362946.1 | [20] |
| Arg/1/00 | Argentina | DQ362947.1 | [20] |
| BRPR/2387/06 | Brazil | HM124483.1 | [21] |
| BRPR/3397/06 | Brazil | HM124484.1 | [21] |
| BRPR/1201/07 | Brazil | HM124491.1 | [21] |
| BRPR/171/08 | Brazil | HM124511.1 | [21] |
| BRPR-175/08 | Brazil | HM124512.1 | [21] |
| BRPR-836/08 | Brazil | HM124513.1 | [21] |
| A1/6621/011 | Italy | KC588902.1 | [22] |
| B1-4702-04 | Italy | KC588903.1 | [22] |
| B2-3817-04 | Italy | KC588904.1 | [22] |
| A2/8909/06 | Italy | KC588906.1 | [22] |
| A2/8908/06 | Italy | KC588907.1 | [22] |
| BR/2/01 | Netherlands | AY295930.1 | [5] |
| NL/6/01 | Netherlands | AY296012.1 | [5] |
| NL/2/02 | Netherlands | AY295965.1 | [5] |
| NL/1/81 | Netherlands | AY295958.1 | [5] |
| NL/4/93 | Netherlands | AY295983.1 | [5] |
| NL/13/00 | Netherlands | AY295950.1 | [5] |
| TN/83-7-7 | United States | EU857608.1 | [8] |
| TN/90/1-40 | United States | EU857605.1 | [8] |
| TN/91/4-51 | United States | EU857598.1 | [8] |
| H0910/163/A | Canada | KF192758 | [23] |
| H0910/193/A | Canada | KF192763 | [23] |
| H0708/303/B | Canada | KF192790 | [23] |
| C0910/1075/B | Canada | KF192803 | [23] |
| HR9722/11 | Croatia | KU375609 | [24] |
| HR19598/11 | Croatia | KU375622 | [24] |
| 0436 | Nepal | MK177102.1 | [25] |
| 1547 | Nepal | MK177105.1 | [25] |
| 6674 | Nepal | MK177124.1 | [25] |

| **HMPV (N) sequence name** | **Country** | **GenBank access number** | **Reference** |
| --- | --- | --- | --- |
| BRPR/2961/06 | Brazil | HM124518.1 | [21] |
| BRPR/2681/06 | Brazil | HM124519.1 | [21] |
| BRPR/2894/06 | Brazil | HM124520.1 | [21] |
| BRPR/2740/06 | Brazil | HM124521.1 | [21] |
| BRPR/1606/07 | Brazil | HM124522.1 | [21] |
| BRPR/1053/07 | Brazil | HM124523.1 | [21] |
| BRPR/1473/07 | Brazil | HM124524.1 | [21] |
| BRPR/2590/07 | Brazil | HM124525.1 | [21] |
| BRPR/2592/07 | Brazil | HM124526.1 | [21] |
| BRPR/2633/07 | Brazil | HM124527.1 | [21] |
| BRPR/1113/08 | Brazil | HM124528.1 | [21] |
| BRPR/1820/06 | Brazil | HM173093.1 | [21] |
| BRPR/2591/07 | Brazil | HM173095.1 | [21] |
| BR/UDI03-300 | Brazil | FJ554881.1 | [26] |
| BR/UDI04-337 | Brazil | FJ554883.1 | [26] |
| BR/UDI04-338 | Brazil | FJ554884.1 | [26] |
| BR/UDI06-421 | Brazil | FJ554885.1 | [26] |
| UY/1/06 | Uruguay | GQ888746.1 | [27] |
| UY/2/06 | Uruguay | GQ888748.1 | [27] |
| UY/5/06 | Uruguay | GQ888749.1 | [27] |
| UY/14/06 | Uruguay | GQ888750.1 | [27] |
| UY/16/06 | Uruguay | GQ888751.1 | [27] |
| UY/7/06 | Uruguay | GQ888755.1 | [27] |
| UY/8/06 | Uruguay | GQ888756.1 | [27] |
| UY/9/06 | Uruguay | GQ888757.1 | [27] |
| UY/15/06 | Uruguay | GQ888759.1 | [27] |
| UY/17/06 | Uruguay | GQ888760.1 | [27] |
| UY/18/06 | Uruguay | GQ888761.1 | [27] |
| NED01-17 | Netherlands | AY355325.1 | [28] |
| NED01-09 | Netherlands | AY355330.1 | [28] |
| BIR01-10 | Netherlands | AY355332.1 | [28] |
| FIN01-10 | Netherlands | AY355334.1 | [28] |
| CAN98-74 | Canada | AY145280.1 | [11] |
| CAN98-15 | Canada | AY145281.1 | [11] |
| CAN98-76 | Canada | AY145282.1 | [11] |
| CAN98-77 | Canada | AY145283.1 | [11] |
| CAN00-15 | Canada | AY145275.1 | [11] |
| Q01-725 | Australia | AY256873.1 | [29] |
| Q01-726 | Australia | AY256874.1 | [29] |
| Q01-629 | Australia | AY256875.1 | [29] |
| Q01-419 | Australia | AF443831.3 | [30] |

**REFERENCES**

1. Lorenzi H, Town C, Halpin R, Nguyen A, Bera J, Fedorova N, et al. Human metapneumovirus. GenBank https://www.ncbi.nlm.nih.gov/bioproject/PRJNA73051 2013;

2. Goya S, Valinotto LE, Tittarelli E, Rojo GL, Jodar SN, Greninger AL, et al. An optimized methodology for whole genome sequencing of RNA respiratory viruses from nasopharyngeal aspirates. 2018;3:1–15.

3. Di Paola N, Cunha M., Oliveira DB., Durigon E, Durigon G., Zanotto PMA. Human metapneumovirus isolate STA755. GenBank https://www.ncbi.nlm.nih.gov/nuccore/MG431250.1 2017;

4. Van Den Hoogen BG, De Jong JC, Groen J, Kuiken T, De Groot R, Fouchier RAM, et al. A newly discovered human pneumovirus isolated from young children with respiratory tract disease. Nat. Med. 2001;7:719–24.

5. Van Den Hoogen BG, Osterhaus DME, Fouchier RAM. Clinical impact and diagnosis of human metapneumovirus infection. Pediatr. Infect. Dis. J. 2004;23:25–32.

6. Graaf M De, Osterhaus ADME, Fouchier RAM, Holmes EC. Evolutionary dynamics of human and avian metapneumoviruses. J. Gen. Virol. 2008;2933–42.

7. Piyaratna R, Tollefson SJ, Williams J V. Genomic Analysis of Four Human Metapneumovirus Prototypes. 2011;160:200–5.

8. Yang C, Wang CK, Tollefson SJ, Piyaratna R, Lintao LD, Chu M, et al. Genetic diversity and evolution of human metapneumovirus fusion protein over twenty years. Virol. J. 2009;6:1–10.

9. Palacios G, Lowenstine LJ, Cran MR, Gilardi KVK, Lukasik-braum M, Kinani J, et al. Metapneumovirus Infection in Wild Mountain Gorillas, Rwanda. Emerg. Infect. Dis. 2011;17:711–3.

10. Dehority W., Kennedy J., Denson J., Schwalm K., Dinwiddie DL. Human metapneumovirus isolate hMPV/USA/AR002/2016. GenBank https://www.ncbi.nlm.nih.gov/nuccore/KY474530.1 2017;

11. Bastien N, Ward D, Van Caeseele P, Brandt K, Lee SHS, McNabb G, et al. Human metapneumovirus infection in the Canadian population. J. Clin. Microbiol. 2003;41:4642–6.

12. Biacchesi S, H M, Skiadopoulos, Boivin G, Hanson TC, Murphy BR, et al. Genetic diversity between human metapneumovirus subgroups. Virology 2003;315:1–9.

13. Wentworth D., Halpin R., Bera J, Lin X, Fedorova N, Tsitrin T, et al. Human metapneumovirus. GenBank https://www.ncbi.nlm.nih.gov/bioproject/PRJNA237298 2014;

14. Xu S, Chen L. Human metapneumovirus isolate hMPV/CHN/18JB00311/2018. GenBank https://www.ncbi.nlm.nih.gov/nuccore/MK087726.1 2019;

15. Song K., Kwak E., Moon K., Lee C., Park K. Human metapneumovirus isolate KUMC-MP. GenBank https://www.ncbi.nlm.nih.gov/nuccore/KF516922.1 2015;

16. Liu W, Xie Z, Qu X, Duan Z, Gao H, Qi Z, et al. Human metapneumovirus isolate CS113. GenBank https://www.ncbi.nlm.nih.gov/nuccore/EF081361.1 2008;

17. Zhu B, Zhong J., Hua L, Xie J., Chen Y. Human metapneumovirus strain HMPVgz01. GenBank https://www.ncbi.nlm.nih.gov/nuccore/GQ153651.1 2009;

18. Liu L, Qian Y, Zhu R, Zhao L, Deng J. Generation of recombinant nucleocapsid protein of human metapneumovirus in baculovirus for detecting antibodies in the Beijing population. Arch. Virol. 2009;155:47–54.

19. Rizotto LS, Scagion GP, Cardoso TC, Simao RM, Caserta LC, Benassi JC, et al. Avian metapneumovirus isolate aMPV-A/chicken/Brazil-SP/669/2003. GenBank https://www.ncbi.nlm.nih.gov/nuccore/MF093139.1 2017;

20. Galiano M, Trento A, Ver L, Carballal G, Videla C. Genetic Heterogeneity of G and F Protein Genes From Argentinean Human Metapneumovirus Strains. J. Med. Virol. 2006;78:631–7.

21. Debur MDC. Metapneumovirus Humano : Diagnóstico Laboratorial E Estudo Clínico-Epidemiológico De Pacientes Hospitalizados E Ambulatoriais Na Cidade De Curitiba Durante Os Anos De 2006 a 2008. 2010;

22. Piralla A, Percivalle E, Baldanti F. Human metapneumovirus in Italy. GenBank https://www.ncbi.nlm.nih.gov/nuccore (accession no.KC588902.1, KC588903.1, KC588904.1, KC588906.1, KC588907.1) 2013;

23. Papenburg J, Carbonneau J, Isabel S, Bergeron MG, Williams J V, Serres G De, et al. Genetic diversity and molecular evolution of the major human metapneumovirus surface glycoproteins over a decade. J. Clin. Virol. 2013;58:541–7.

24. Jagusic M, Slovic A, Forcic D, Ljubin Sternak S, Mlinaric-Galinovic G. Human metapneumovirus (HMPV) strains detected in Croatia, 2011-2014. Unpubl. GenBank 2016;

25. Kuypers J, Perchetti GA, Chu HY, Magaret AS, Katz J, Khatry SK, et al. Direct Submission. Unpubl. GenBank

26. Carneiro BM, Yokosawa J, Arbiz J, Costa LF, Mirazo S, Nepomuceno LL, et al. Detection of All Four Human Metapneumovirus Subtypes in Nasopharyngeal Specimens from Children with Respiratory Disease in Uberlaˆ ndia, Brazil. J. Med. Virol. 2009;5581:1814–8.

27. Pizzorno A, Masner M, Medici C, Sarachaga MJ, Rubio I, Mirazo S, et al. Molecular Detection and Genetic Variability of Human Metapneumovirus in Uruguay. J. Med. Virol. 2010;82:861–5.

28. Maertzdorf J, Wang CK, Brown JB, Quinto JD, Chu M, Graaf M De, et al. Real-Time Reverse Transcriptase PCR Assay for Detection of Human Metapneumoviruses from All Known Genetic Lineages. J. Clin. Microbiol. 2004;42:981–6.

29. Mackay IM, Bialasiewicz S, Waliuzzaman Z, Chidlow GR, Fegredo DC, Laingam S, et al. Use of the P Gene to Genotype Human Metapneumovirus Identifies 4 Viral Subtypes. J. Infect. Dis. 2004;190:1913–8.

30. Mackay IM, Jacob KC, Woolhouse D, Waller K, Syrmis MW, Whiley DM, et al. Molecular Assays for Detection of Human Metapneumovirus Molecular Assays for Detection of Human Metapneumovirus. J. Clin. Microbiol. 2003;41:100–5.
