# Supplementary material for: Human Metapneumovirus: Epidemiology and genotype diversity in children and adult patients with respiratory infection in Córdoba, Argentina
Source: PLoS One. 2020 Dec 28;15(12):e0244093. doi: 10.1371/journal.pone.0244093 (PMC7769284; doi:10.1371/journal.pone.0244093)
Supplement: S2 Table — (DOC) [file pone.0244093.s002.doc]

**S2** Table. Local sequences of N gene shorter than 200 bp.

| **Identification** | **Nucleotide sequence** |
| --- | --- |
| **Cordoba/ARG/2590/2011** | GAAACAAAAATTGCTAGATCCTTCTATGACTTATTTGAACAAAAAGTGTATCACAGAAGTTTGTTCATTGAGTATGGCAAAGCATTAGGCTCATCCTCTACAGGCAGCAAAGCAGAAAGTTTATTCGTCAACATTTTCATGCAAGC |
| **Cordoba/ARG/2629/2011** | GGAAACCACAAATTGCTAGATCCTTCTATGACTTATTTGAACAAAAAGTGTATCACAGAAGTTTGTTCATTGAGTATGGCAAAGCATAGGCTCATCCTCTACAGGCAGCAAAGCAGAAAGTTTATTCGTCAACATATTCATGCAA |
| **Cordoba/ARG/2708/2011** | GGGAGAGGAATGAAGATTTTCTATGACTTATTTGAACAAAAAGTGTATCACAGAAGTTTGTTCATTGAGTATGGCAAAGCATTAGGCTCATCCTCTACAGGCAGCAAAGCAGAAAGTTTATTCGTCAACATATTCATGCAAGCTTATGGGGCCGGTCAAACAATGAGG |
| **Cordoba/ARG/2732/2011** | GGAACCAAAAATGCTAGATCCTTCTATGACTTATTTGAACAAAAAGTGTATCACAGAAGTTTGTTCATTGAGTATGGCAAAGCATTAGGCTCATCCTCTACAGGCAGCAAAGCAGAAAGTTTATTCGTCAACATATTCATGCAA |
| **Cordoba/ARG/2755/2011** | ATACCAAAAATTGCTAGATCCTTCTATGACTTATTTGAACAAAAAGTGTATCACAGAAGTTTGTTCATTGAGTATGGCAAAGCATTAGGCTCATCCTCTACAGGCAGCAAAGCAGAAAGTTTATTCGTCAACATATTCATGCAAGC |
| **Cordoba/ARG/2840/2011** | GTTTTCCAAAAAGATAGATCCTTCTATGACTTATTTGAACAAAAAGTGTATCACAGAAGTTTGTTCATTGAGTATGGCAAAGCATTAGGCTCATCCTCTACAGGCAGCAAAGCAGAAAGTTTATTCGTCAACATATTCATGCAA |
| **Cordoba/ARG/2899/2011** | AAAAATGATAGATCCTTCTATGACTTATTTGAACAAAAAGTGTATCACAGAAGTTTGTTCATTGAGTATGGCAAAGCATTAGGCTCATCCTCTACAGGCAGCAAAGCAGAAAGTTTATTCGTCAACATATTCATGCAA |
